# Frequency and phenotype of thalamic aphasia

**DOI:** 10.1007/s00415-021-10640-4

**Published:** 2021-06-08

**Authors:** Ida Rangus, Merve Fritsch, Matthias Endres, Birgit Udke, Christian H. Nolte

**Affiliations:** 1 Department of Neurology with Experimental Neurology, Charité - Universitätsmedizin Berlin, Corporate Member of Freie Universität Berlin, Humboldt-Universität zu Berlin and Berlin Institute of Health, Campus Benjamin Franklin, Hindenburgdamm 30, 12203 Berlin, Germany; 2 Department of Psychiatry, Charité-Universitätsmedizin Berlin, Campus Charité Mitte, Berlin, Germany; 3 Center for Stroke Research Berlin, Berlin, Germany; 4 ExcellenceCluster NeuroCure, Berlin, Germany; 5 German Center for Neurodegenerative Diseases (DZNE), Berlin, Germany; 6 German Centre for Cardiovascular Research (DZHK), Berlin, Germany; 7 Department of Audiology and Phoniatrics, Charité-Universitätsmedizin Berlin, Berlin, Germany

**Keywords:** Thalamus, Stroke, Aphasia, Language

## Abstract

**Background:**

Aphasia is a recognized presenting symptom of thalamic lesions. Little is known regarding its frequency and phenotype. We examined the frequency of thalamic aphasia following Isolated Acute unilateral ischemic Lesions in the Thalamus (IALT) with respect to lesion location. Furthermore, we characterized thalamic aphasia according to affected language domains and severity.

**Methods:**

Fifty-two patients with IALT were analyzed [44% female, median age: 73 years (IQR: 60–79)]. Lesion location was determined using 3-Tesla magnetic resonance imaging and categorized as anterior, posterior, paramedian or inferolateral. Standardized language assessment was performed using the validated Aphasia checklist (ACL) directly after symptom onset. Aphasia was defined as an ACL sum score of < 135 (range: 0–148).

**Results:**

Of 52 patients, 23 (44%) fulfilled the ACL diagnostic criteria for aphasia, including nearly all lesion locations and both sides. The average ACL sum score was 132 ± 11 (range: 98–147). Aphasia was characterized by deficits within domains of complex understanding of speech and verbal fluency. Patients with left anterior IALT were most severely affected, having significantly lower ACL scores than all other patients (117 ± 13 vs. 135 ± 8; *p* < 0.001). In particular, aphasia in patients with left anterior IALT was characterized by significantly worse performance in the rating of verbal communication, verbal fluency, and naming (all *p* ≤ 0.001).

**Conclusion:**

Aphasia occurs in almost half of patients with focal thalamic lesions. Thalamic aphasia is not confined to one predefined thalamic lesion location, but language deficits are particularly pronounced in patients with left anterior IALT presenting with a distinct pattern.

## Introduction

The involvement of subcortical structures in language has lately gained much scientific attention. In contrast to the outdated assumption that language processing only occurs within the frontal, temporal and parietal cortex of the language-dominant hemisphere, the current understanding of the neurophysiological organization of language is that of a cortico-subcortical language network, involving the thalamus [1, 2]. In addition to filtering, modulating, and integrating afferent signals before reaching the cerebral cortex, the thalamus is also involved in the regulation of the level of consciousness and sleep and plays an important role in language and cognition [3]. Rather than working in isolation from other cerebral language areas, the thalamus is believed to selectively engage frontal, parietal, and temporal cortical areas necessary to perform language tasks [1]. The term “thalamic aphasia” is widely accepted. It is considered to result from “diaschisis”, which implies that anatomically intact cortical structures involved in language are impaired through disruption of cortico-subcortical connections caused by thalamic lesions [4].

So far, our knowledge on “thalamic aphasia” is based on case reports and case series with heterogenous study populations (e.g. ischemic and hemorrhagic stroke, Parkinson’s disease with deep brain stimulation) and various language assessment methods with different time intervals between symptom onset and language assessment [4–6]. Some of these studies were limited by lacking details on the affected thalamic subregions. Other studies even excluded right-sided lesions. Previous estimations of the frequency of thalamic aphasia varied greatly from 12 to 88%. The variation of frequency mostly depended on the type of language assessment methods. Studies using less detailed examinations reported lower frequencies [4, 5, 7–10]. More sophisticated language assessment methods acknowledge that communication impairments extend beyond verbal deficits and that aphasic symptoms might not result solely from a faulty language system. For example, smooth communication also depends on the integrity of executive function skills. Executive functions come into play when an individual is involved in a complex, novel activity [11]. Therefore, depicting affected language domains in “thalamic aphasia” is of special interest. Distinctive language impairment patterns have been described in known cases of “thalamic aphasia” [4]. Witte et al. suggested that the following characteristics would be typical: fluent output, normal or mildly impaired comprehension skills and repetition, moderate to severe anomia, hypophonia/articulation difficulties, and reduction of spontaneous speech [4]. Other authors also reported semantic and phonemic paraphasic errors with occasional neologisms and perseverations [3, 8, 12, 13]. Overall, severity has been classified as mild with patients recovering fast [4, 14].

Some authors have particularly assigned language impairment to lesions in the left anterior thalamus [7, 15]. However, aphasia has been observed after lesions in anterior, paramedian, posterior, and inferolateral thalamic subregions [3]. Thus, it is not unequivocal, which thalamic subregions are associated with language.

Systematic data on language performance in patients with acute focal thalamic lesions is missing. The aim of this study was to examine the frequency of thalamic aphasia after Isolated Acute unilateral ischemic Lesions in the Thalamus (IALT) with respect to lesion location. Furthermore, we sought to characterize thalamic aphasia according to affected language domains and severity with a standardized, uniform and sensitive assessment tool using the validated Aphasia check list (ACL).

## Materials and methods

### Participants

**Fig. 1:**
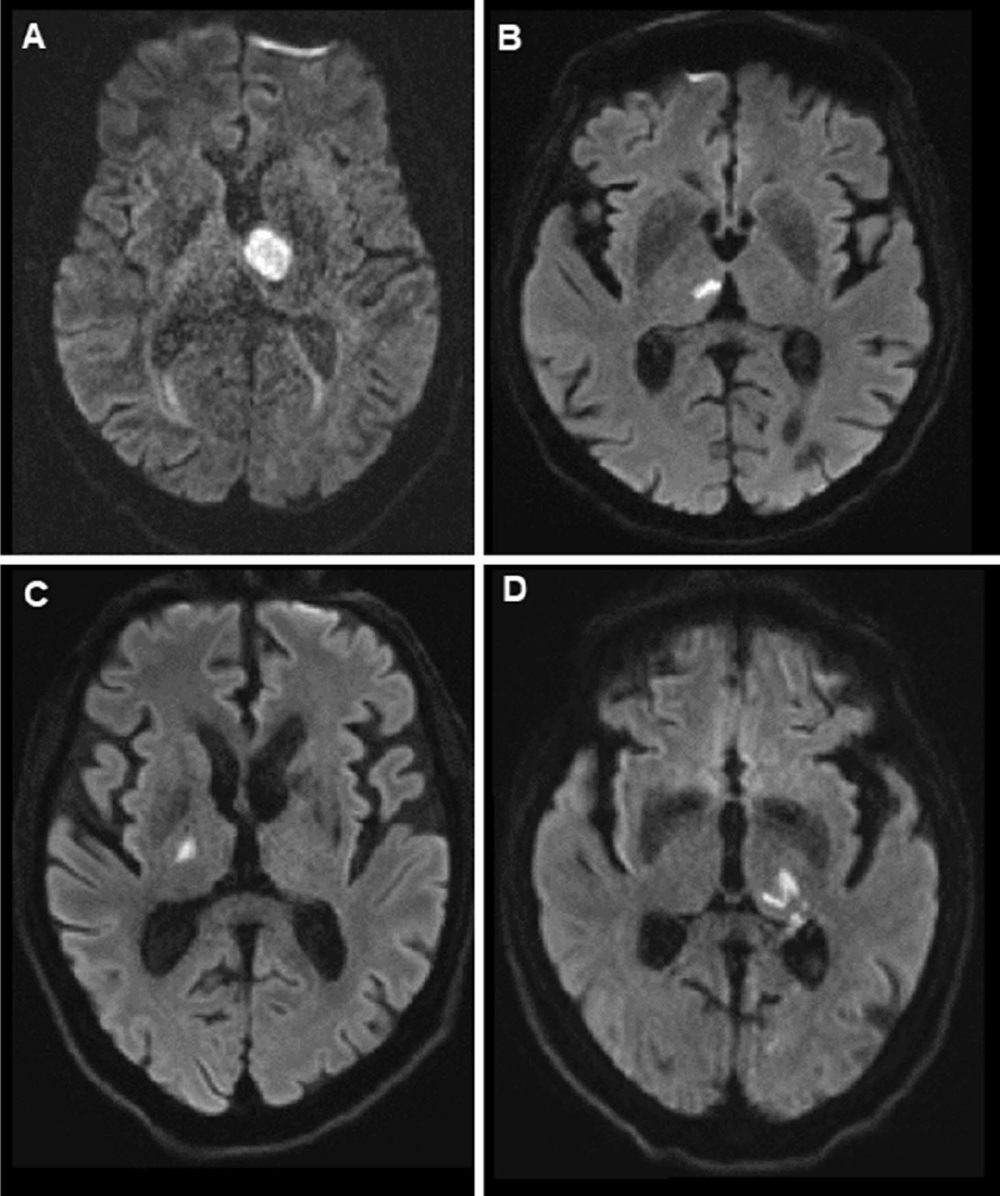
Examples of IALT localizations in the DWI sequence: **A** left anterior **B** right paramedian **C** right inferolateral **D** left posterior

We enrolled 52 patients who were consecutively admitted to the Stroke Unit of the Department of Neurology at Charité - Universitätsmedizin Berlin, Campus Benjamin Franklin between July 2017 and June 2020 undergoing magnetic resonance imaging (MRI) proving Isolated Acute unilateral ischemic Lesions in the Thalamus (IALT) who received ACL. Patients were either German native speakers or had a very good knowledge of the German language. Stroke severity was assessed using the National Institutes of Health Stroke Scale (NIHSS). Patients with bilateral thalamic lesions, hemorrhagic strokes or additional acute lesions in other cerebral arterial territories and patients with chronic strokes in the territory of the left middle cerebral artery were excluded. Further exclusion criteria were pre-existing aphasia and neurological or psychiatric comorbidities such as severe depression, dementia, and substance abuse (Fig. 1).

### Imaging methods

Patients underwent a standard stroke imaging protocol using susceptibility-weighted imaging (SWI), diffusion-weighted imaging (DWI) and fluid-attenuated inversion recovery (FLAIR) MRI sequences, performed on a 3-Tesla Siemens Magnetom Trio scanner (Siemens Medical Solutions, Erlangen, Germany). Acute thalamic strokes were diagnosed in the DWI sequence with 2.5 mm slice thickness. Images were analyzed by two neuroradiologists, respectively. Patients were grouped according to the affected vascular territory within the thalamus as previously described (anterior, paramedian, inferolateral and posterior) [3, 16–18].

### Language assessment

Language assessment was conducted by trained speech therapists blinded for the thalamic lesion location, using the German Aphasia checklist (ACL). Time between symptom onset and language assessment was recorded. The ACL was validated in a population of aphasic patients and healthy subjects using Aachen Aphasia Test [19]. It consists of seven subtests: (I) automatic speech (naming days of the week and counting from 1 to 15), (II) verbal instructions (following instructions to perform easy actions, e.g. knocking on the table), (III) color-figure test/complex understanding of speech (the patient is requested to show items with different shapes, sizes and colors on a table with instructions of increasing complexity), (IV) verbal fluency/word generation tasks (letter fluency task: generating as many words as possible with the initial letter B, semantic fluency task: generating as many items as possible that can be bought in a supermarket; one minute per item), (V) specific linguistic abilities (six tasks that asses important linguistic abilities, such as confrontation naming, reading aloud, reading comprehension, auditory comprehension, writing to dictation and repeating words and sentences; in addition, the processing of “pseudowords” is scored separately), (VI) rating of verbal communication (assessment of patients’ “everyday” communication skills as evaluated by a speech therapist), (VII) number processing (reading numbers aloud, writing numbers to dictation and repeating numbers). Additionally, spontaneous speech is rated as fluent or non-fluent by speech therapists.

Raw scores achieved in separate tasks can each be transformed into degrees of impairment ranging from 0 to 3, where 3 indicates no language disorder, 2 indicates mild or residual aphasia, 1 indicates moderate language disorder and 0 means severely disturbed language production and reception. This way, ACL depicts an individual language profile and does not categorize impaired language modalities into syndromes.

Furthermore, the severity of aphasia, in general, can be determined with an aphasia sum score. It is generated by adding up raw scores of the most important subtests (color-figure test, verbal fluency tasks, confrontation naming, reading comprehension, auditory comprehension, writing to dictation, and repeating words and sentences). The highest possible score is 148 points. Sum scores below 135 and impairment in at least one language comprehension subtest and one language production subtest indicate aphasia.

The ACL also entails a short cognitive test that examines working memory, attention, and reasoning [19]. This part of the ACL was not subject of the current study.

### Statistics

Statistical analyses were carried out using IBM SPSS Statistics, Version 25. Nominal data were analyzed using chi-square test. The comparisons of ACL sum scores and scores in separate subtests between left and right IALT as well as left anterior IALT and non-left-anterior IALT were performed using Mann–Whitney *U* test. Due to small numbers, two-tailed exact significance was reported. The results were considered significant, where *p* value was < 0.05.

## Results

### Demographic data

Overall, 44% (23/52) of the patients were female. The median age was 73 years (IQR: 60–79). The median NIHSS score on admission was 2 (IQR: 1–4). Leading cardiovascular risk factors in our study population were arterial hypertension with 62% of the patients, followed by smoking (31%), diabetes mellitus (19%) and atrial fibrillation (14%). All patients were alert while ACL was performed. The median time between symptom onset and ACL testing was 2 days (IQR: 2–3). The average number of days from stroke onset to language examination was evenly distributed among different thalamic subregions, ruling out confounding effects.

### Lesion location within the thalamus

IALT was observed on the left side in 31 patients and on the right side in 21 patients. The majority of IALTs were located in the left and right inferolateral thalamus with 18 and 16 patients, respectively. Lesions in the posterior thalamus were the least common. A detailed listing of lesion location is shown in Table 1.

**Table 1:** Stroke location within the thalamus

	Left thalamus (*n* = 31) Number of patients (%)	Right thalamus (*n* = 21) Number of patients (%)	(*n* = 52)
Anterior	8 (15%)	3 (6%)	11 (21%)
Paramedian	4 (8%)	2 (4%)	6 (12%)
Inferolateral	18 (34%)	16 (31%)	34 (65%)
Posterior	1 (2%)	0	1 (2%)

### Frequency and severity of aphasia in thalamic stroke

Of 52 patients, 23 (44%) had an ACL sum score < 135 and an impairment in at least one language comprehension subtest and one language production subtest and thereby fulfilled diagnostic criteria for aphasia as defined by the ACL. All patients underwent ACL, but ACL sum score was not reported for 3 patients.

The mean ACL-score in the whole study population was 132 ± 11 (range: 98–147) (*n* = 49), indicating mild language impairment in IALT in general. The frequency of aphasia per se did not differ significantly between the affected side (left vs. right: 15/31 vs. 8/21, *p* = 0.573), nor did the mean ACL-score (left vs. right: 130 ± 11 vs. 135 ± 9, *p* = 0.103). Furthermore, the higher frequency of aphasia in left anterior IALT (6/8 = 75%) did not reach statistical significance when compared to all other IALT locations (17/44 = 39%), *p* = 0.118. Nevertheless, the mean ACL score was significantly lower in patients with left anterior IALT compared to patients with non-left-anterior IALT (117 ± 13 vs. 135 ± 8, *p* < 0.001). Table 2 shows the mean ACL sum score for each affected thalamic region separately.

**Table 2:** Frequency of aphasia and mean ACL score according to separate thalamic subregions

Thalamic subregion	Number of patients	Number of aphasic patients (ACL < 135)	ACL sum score^a^ (mean ± SD), lower numbers indicate worse aphasia
Left anterior	8	6	117 ± 13
Left paramedian	4	0	138 ± 3
Left inferolateral	18	8	134 ± 6
Left posterior	1	1	123
Right anterior	3	2	128 ± 11
Right paramedian	2	1	136 ± 5
Right inferolateral	16	5	136 ± 9

*ACL* Aphasia check list, *SD* standard deviation

^a^ACL sum score was missing for 3 patients (1 × left anterior, 1 × left paramedian, 1 × left inferolateral)

### Characteristics of thalamic aphasia

We separately analyzed the performance in each language modality. Performance in the spontaneous speech was not reported for seven patients. Furthermore, verbal communication skills and the performance in automatic speech was not specifically reported for five patients.

In general, language deficits were mild. Of 45 patients, whose spontaneous speech was examined, 43 (96%) showed fluent spontaneous speech. Deficits were most commonly present in verbal fluency, especially in letter fluency task, with impairments being present in 67% (35/52) of patients as well as in the complex understanding of speech and semantic fluency task, which were both affected in 60% (31/52) of patients. Problems with naming were only seen in 19% (10/52) of patients. Auditory and verbal comprehension were not impaired. Neither was repeating words, reading and writing as well as processing numbers. In addition to highlighting impaired language modalities, Table 3 shows the degree of their impairment.

**Table 3:** Comparison of the degree of impairment in each ACL subitem between left and right IALT

ACL subitem	All (*n* = 52) Degree of impairment median [IQR]	Left IALT (*n* = 31) Degree of impairment median [IQR]	Right IALT (*n* = 21) Degree of impairment median [IQR]	*p* value^1^
I Automatic speech	3 [3–3]^a^	3 [3–3]^b^	3 [3–3]	0.321
II Verbal instructions	3 [3–3]	3 [2.25–3]	3 [2.5–3]	0.872
III Color-figure test/complex understanding of speech	2 [2–3]	2 [2–2.75]	3 [2–3]	0.209
IV Verbal fluency				
Letter fluency task	2 [1–3]	2 [1–2.75]	2 [1–3]	0.110
Semantic fluency task	**2 [1–3]**	**2 [1–2.75]**	**3 [1–3]**	**0.042**
V specific linguistic abilities				
Confrontation naming	**3 [3–3]**	**3 [2–3]**	**3 [3–3]**	**0.028**
Reading aloud	3 [3–3]	3 [3–3]	3 [3–3]	0.158
Reading comprehension	3 [2–3]	3 [2–3]	3 [2–3]	0.984
Auditory comprehension	3 [3–3]	3 [3–3]	3 [3–3]	0.889
Writing to dictation	3 [3–3]	3 [3–3]	3 [3–3]	0.613
Repeating	3 [3–3]	3 [3–3]	3 [3–3]	1.000
Reading pseudowords	3 [3–3]	3 [3–3]	3 [3–3]	1.000
Writing pseudowords	3 [3–3]	3 [2–3]	3 [3–3]	0.720
Repeating pseudowords	3 [3–3]	3 [2.25–3]	3 [3–3]	0.354
VI Rating of verbal communication	**3 [3–3]**^a^	**3 [2–3]**^b^	**3 [3–3]**	**0.041**
VII Number processing				
Reading numbers	3 [3–3]	3 [3–3]	3 [3–3]	1.000
Writing numbers	3 [3–3]	3 [3–3]	3 [3–3]	0.264
Repeating numbers	3 [3–3]	3 [3–3]	3 [3–3]	1.000

Degree of impairment: 3 = no language disorder, 2 = mild or residual aphasia, 1 = moderate language disorder, 0 = severely disturbed language production and reception

^a^*n* = 47

^b^*n* = 26

^1^Mann-Whitney *U* test; two-tailed exact significance bold values represent significant differences of the degree of impairment in ACL subitems between the left and right IALT

### Comparison of affected language modalities between left and right IALT

Left-sided IALT was significantly more commonly associated with impaired verbal communications skills (*p* = 0.041), semantic fluency task (*p* = 0.042) and naming (*p* = 0.028) than right-sided IALT. There were no significant differences of language performance in other subscales. A comparison of the degree of impairment in separate language modalities between the left and right IALT is shown in Table 3.

### Characteristics of aphasia in patients with left anterior IALT

Since patients with left anterior IALT had the lowest ACL sum score compared to patients with all other IALT locations, we conducted a separate analysis of the frequency and characteristics of aphasia in these patients. Six of eight patients (75%) with left anterior IALT showed aphasia. All patients in this group showed deficits in verbal fluency, including both letter and semantic fluency tasks. The comparison of the degree of impairment in separate subitems of the ACL revealed significantly worse performance of the patients with left-anterior IALT in the domains of verbal communication skills, semantic and letter fluency, naming (all *p* ≤ 0.001), as well as a complex understanding of speech (*p* = 0.017) and automatic speech (*p* = 0.022), compared to patients with non-left-anterior IALT. Detailed results are shown in Table 4.

**Table 4:** Comparison of the degree of impairment in each ACL subitem between left anterior and non-left-anterior IALT

ACL subitem	Left anterior IALT (*n* = 8) Degree of impairment median [IQR]	Non-left-anterior IALT (*n* = 44) Degree of impairment median [IQR]	*p* value^1^
I Automatic speech	**2.5 [1.25–3]**^a^	**3 [3–3]**^b^	**0.022**
II Verbal instructions	2 [0.5–2.75]	3 [3–3]	0.070
III Color-figure test/complex understanding of speech	**1 [1–1.75]**	**2 [2–3]**	**0.017**
IV Verbal fluency			
Letter fluency task	**0 [0–0.75]**	**2 [1–3]**	**< 0.001**
Semantic fluency task	**1 [0.25–1]**	**2 [1–3]**	**0.001**
V Specific linguistic abilities			
Confrontation naming	**1 [0.25–1]**	**3 [3–3]**	**< 0.001**
Reading aloud	3 [3–3]	3 [3–3]	1.000
Reading comprehension	2 [1.25–2.75]	3 [2–3]	0.738
Auditory comprehension	2.5 [1.25–3]	3 [3–3]	0.076
Writing to dictation	3 [1.5–3]	3[3–3]	0.749
Repeating	3 [1.5–3]	3 [3–3]	0.154
Reading pseudowords	2.5 [2–3]	3 [3–3]	0.094
Writing pseudowords	2.5 [2–3]	3 [3–3]	0.366
Repeating pseudowords	2.5 [1.25–3]	3 [3–3]	0.452
VI Rating of verbal communication	**1 [1–1]**^a^	**3 [3–3]**^b^	**< 0.001**
VII number processing			
Reading numbers	3 [3–3]	3 [3–3]	1.000
Writing numbers	3 [2.25–3]	3 [3–3]	0.401
Repeating numbers	3 [3–3]	3 [3–3]	1.000

Degree of impairment: 3 = no language disorder, 2 = mild or residual aphasia, 1 = moderate language disorder, 0 = severely disturbed language production and reception

^a^*n* = 4

^b^*n* = 43

^1^Mann-Whitney *U* test; two-tailed exact significance bold values represent significant differences of the degree of impairment in ACL subitems between left anterior IALT and non-left anterior IALT

## Discussion

The aim of our study was to assess the frequency and phenotype of aphasia after acute thalamic lesions by utilizing the ACL. Our results present a major contribution to the topic of “thalamic aphasia”, as we are the first, to our knowledge, to perform a systematic, standardized, and uniform language assessment in a large and well-defined cohort of patients with focal thalamic lesions. 3-Tesla MRI allowed high specificity, proof of acuity, and high spatial resolution.

The frequency of aphasia as defined by the ACL was 44%, which is within the range of previous reports and particularly similar to previously reported higher frequencies in studies that employed elaborate language assessment tools. [4, 5, 7–10]. Aphasia was especially severe and frequent in left anterior thalamic infarction but also seen, although less severe, in right anterior and left inferolateral thalamic infarction. Since aphasia was found in almost half of the patients with acute focal thalamic lesions, aphasia screening appears appropriate for all patients with acute thalamic lesions to timely recognize those who might require and benefit from speech therapy. The mean ACL sum score in our study was close to the cut-off defining presence of aphasia (< 135), indicating only mild aphasic symptoms in most patients. Sensitive testing improved the likelihood of identifying subtle language impairments, which might partially account for the unexpected high frequency of aphasia in right-sided lesions. Such subtle aphasic symptoms that are considered typical of thalamic lesions may remain unnoticed if thorough language testing does not take place. Our study did not include follow-up examinations. Therefore, we cannot account for the frequency of aphasia in the intermediate phase post-stroke, where it is expected to be lower, since thalamic aphasia has mostly been described as transient before [4].

The phenotype of “thalamic aphasia” found in our study corresponds to earlier reports to some extent. As previously reported, we mainly found unimpaired comprehension and repetition. However, most of our patients did not show reduced spontaneous speech, although this had been reported previously [4]. Language deficits were most notably observed in verbal fluency tasks (letter and semantic fluency), where lexical retrieval, processing speed, cognitive flexibility (choosing strategies for word retrieval) und imagination is challenged. Interestingly, this domain was unimpaired in thalamic aphasias in De Witte’s work (who had listed it under the term “fluency”) [4]. Here, we show that systematic language assessment not only confirms impaired verbal fluency in patients with thalamic lesions but even emphasizes it as an integral part of thalamic aphasia. Supporting evidence comes from Kuljic-Obradovic and Crosson who previously stressed the importance of the dominant thalamus for lexical-semantic processing and word retrieval [12, 15]. Impaired complex understanding of speech was another significant finding in our study. This item assesses auditory comprehension for abstract verbal content and, at least with more complex tasks, verbal short-term memory [19]. Analogous to our findings, auditory comprehension was previously shown to deteriorate with increasing length and complexity in patients with stroke in the left anterior thalamus [20]. Due to their complexity, verbal fluency and complex understanding of speech can be considered as higher-order language skills. Some authors acknowledge the involvement of the thalamus in language to be especially relevant in such higher-order language domains that require a higher level of alertness [21, 22]. Moreover, verbal fluency depends both on verbal abilities and executive functions [23, 24]. Therefore, impaired verbal fluency may well be a symptom of dysexecutive syndrome. Further differentiation between primary language impairment and involvement of higher cortical functions, such as executive functions is not possible in our patients, because neither additional language assessment techniques nor separate tests of executive functions were performed. We do however argue that strictly adhering to the ACL criteria, which require deficits in at least one productive and one receptive language domain in addition to performance under a given cut-off score, aphasia can be confirmed in patients that fulfilled these criteria. Our findings support the idea of a language network involving subcortical and frontal, parietal, and temporal cortical areas and associated cognitive functions. For better understanding of the language organization, implementing a more thorough neuropsychological assessment of other areas of cognition should be considered in future research. Articulation and prosody were not specifically reported with the ACL, thus data on hypophonia and articulation deficits, previously described as being pathognomonic of thalamic aphasia, cannot be accounted for in our study. Finally, only 19% of our patients showed impaired naming, which is in contrast to previous reports suggesting anomia to be a frequent finding in thalamic aphasia [4]. Only patients with left anterior IALT considered separately showed severe anomia in our study.

In sum, thalamic aphasia can be described as mild, with fluent spontaneous speech and impairments within higher-order language skills such as complex-understanding of speech and verbal fluency as well as unimpaired comprehension and repetition. In addition, severely impaired naming is assigned to the left anterior IALT in particular. Language performance was worst in patients with left anterior IALT in our study. Previously, Fritsch et al. also found lesions in the left anterior thalamus to be strongly associated with aphasia [7]. Despite the aforementioned clues for the importance of the left anterior thalamus in language processing, its exact role herein is yet to be identified. So far, functional imaging studies showed bilateral thalamic activation in tasks involving verbal fluency and naming, with pronounced activation on the left, giving some evidence for the lateralization of the language on the thalamic level [25, 26]. Beyond assigning the left thalamus an important role in language processing, functional imaging studies have not yet succeeded in identifying specific thalamic subregions involved. Attempts at identifying reciprocal connections between thalamic nuclei and overlying cortical regions (mostly conducted in primates) revealed projections between the ventral anterior nucleus of the thalamus and Broca’s area [27] as well as between the ventrolateral nucleus and posterior parietal and prefrontal cortex [28–30]. Future studies should continue exploring correlations between clinical features of thalamic aphasia and related thalamic regions in the context of thalamo-cortical networks.

### Limitations and strengths

Strengths of this study include high-resolution imaging with 3-Tesla MRI and a uniform, standardized language testing using the validated ACL in the largest population of patients with acute focal thalamic lesions so far. Nevertheless, subgroups with posterior and paramedian IALT had very little patients, which made it difficult to generalize data on these regions and overall to compare different thalamic regions. Because of small number, statistical power is limited, and type-2-error has to be taken into account. Second, division of the thalamus into four different regions was based on previously proposed arterial territories with no respect to possible vascular variations. Third, our study only focused on aphasia, as diagnosed using the ACL. However, thalamic lesions have been associated with broader neuropsychological deficits (such as memory impairment or dysexecutive syndrome) which might additionally influence language. Although giving a detailed phenomenology, our analysis on the origin of these language deficits remains limited to some extent. We suggest adding a detailed (non-verbal) assessment of other areas of cognition in future studies to further delineate other cognitive domains involved. Fourth, follow-up examination was missing, so the course of thalamic aphasia cannot be accounted for in our study. Finally, there was no data on the level of education and handedness in our study population. The latter made it impossible to adjust our results according to the language-dominant hemisphere.

### Summary

In this large sample with well-defined isolated acute lesions of the thalamus and standardized language testing, aphasic symptoms were prevalent in almost half of patients. The presence of aphasia was not clearly confined to the left or right side but was markedly pronounced in patients with left anterior lesion location. Thalamic aphasia was characterized by impaired verbal fluency and complex understanding of speech, but intact comprehension and repetition. Left anterior thalamic aphasia additionally stood out with impairment of verbal communication skills and naming.

## Abbreviations

ACL: Aphasia checklist
DWI: Diffusion-weighted imaging
FLAIR: Fluid-attenuated inversion recovery
IALT: Isolated Acute unilateral ischemic Lesions in the Thalamus
MRI: Magnetic resonance imaging
NIHSS: National Institutes of Health Stroke Scale
SWI: Susceptibility weighted imaging

## Declarations

### Availability of data and materials

Anonymized data from the present study will be shared on reasonable request from any qualified researcher for well-defined research questions. Please contact the corresponding author.
